# Occupational cancer burden in developing countries and the problem of informal workers

**DOI:** 10.1186/1476-069X-10-S1-S10

**Published:** 2011-04-05

**Authors:** Vilma Sousa Santana, Fatima Sueli Neto Ribeiro

**Affiliations:** 1 Program of Environmental and Workers Health, Institute of Collective Health, Federal University of Bahia, Brazil; 2 Social Nutrition Department, Rio de Janeiro State University, Brazil

## Abstract

Most workplaces in developing countries are “informal”, i.e. they are not regularly surveyed/inspected and laws for workers’ protection are not implemented. Research on occupational risks in informal workplaces and the related cancer burden is needed. The results of studies addressing exposures among informal workers are difficult to generalize because of the specificities of social contexts, and study populations are small. The estimation of the burden of cancers attributable to occupational exposures is also made difficult by the fact that occupational cancers are usually clinically indistinguishable from those unrelated to occupation.

## Article

According to WHO guidelines, cancer prevention requires information on morbidity and mortality, identification of the most relevant causes and risk factors, where carcinogens are, how individuals become exposed, which are the most vulnerable groups, and what works better to eliminate or reduce the number of exposed or exposure levels [1]. However, available health information remains a challenge in most countries, **particularly in African and Asian countries**[2]. For instance, a recent study on mortality information systems of all American countries shows that only 39.6% were considered as good, and no data was available for 16 countries [3]. Data on cancer morbidity are likely to be worse. In 2006, population-based cancer registries covered only 21% of the world population. Their quality and coverage were uneven across regions, with developing countries having a less favorable situation. Only 11% of the population were covered in Africa, 8% in Asia, while almost all inhabitants (99%) of North America could be reached by cancer registries [4]. Lack of reliable data is an obstacle to establish cancer prevention as a priority in public policies, particularly in poor regions.

It is well established that individual habits such as **smoking and alcohol consumption are major** contributors to cancer burden [1]. However workplaces continue to be a substantial source of carcinogen exposures [5], also including psychosocial stressors that can mediate exposure to relevant cancer risk factors such as smoking and alcohol consumption. The work environment could be of particular relevance in developing countries where cancer mortality is growing [2]. Enforcement of hazard control in workplaces is weak and workers organizations are not strong enough to ensure compliance with standards required for healthy and safe work environments. A study carried out in Brazil with firms undergoing labor inspections revealed that the great majority (92.9%) does not comply with safety norms, particularly collective preventive practices (71.4%) against hazards in the workplace [6] .

This situation can be aggravated in the informal economy where firms are out of State control, not reached by the enforcement of labor regulations concerning workers’ health and safety. Informal economy is increasing in developed and developing countries, and can represent more than 60% of labor force, especially in rural areas [7]**.** Firms from the informal economy are usually non-registered small businesses and are not targeted by labor or health and safety inspections, commonly workers are not unionized, or are poorly organized, and have limited power to make pressure for safe and healthy workplaces. Therefore it is not plausible that they have the same levels of carcinogen exposures when compared to workers from firms in the formal economy. Table 1 shows estimates of the magnitude of informal economy by sex in countries from low and middle income regions. It can be seen that informal economy represents the majority of labor force in several countries, mainly those with low income [8]. However, there are very few studies on occupational hazards or diseases addressing informal workers, or health differences between formal and informal workers. There are reports of increased risks due to work-related stress, and common psychological disorders, among informal workers when compared to formal workers [9], but little is known about carcinogen exposures or cancer occurrence among informal jobs.

**Table 1:** Proportion of informal employment in non-agricultural workforce, according to sex (1994-2000).

Region/Country^a^	Informal Employment^b^		
	
	Women (%)	Men (%)	Total (%)
**Low income**	89.6	71.0	78.8
**Sub-Saharan Africa**			
Benin	97.0	87.0	93.0
Chad	95.0	60.0	74.0
Guinea	87.0	66.0	72.0
Kenya	83.0	59.0	72.0
**Ásia**			
Índia	86.0	83.0	83.0
**Middle income**	53.4	52.8	53.3
**North África**			
Tunísia	39.0	53.0	50.0
Algeria	41.0	43.0	43.0
Morocco	47.0	44.0	45.0
Egypt	46.0	57.0	55.0
**Sub-Saharan Africa**			
South Africa	58.0	44.0	51.0
**Latin America**			
Bolivia	74.0	55.0	63.0
Brazil	67.0	55.0	60.0
Chile	44.0	31.0	36.0
Colômbia	44.0	34.0	38.0
Costa Rica	44.0	48.0	42.0
Dominican Republica	50.0	47.0	48.0
El Salvador	69.0	46.0	57.0
Guatemala	69.0	47.0	56.0
Honduras	65.0	74.0	58.0
Mexico	55.0	54.0	55.0
Venezuela	47.0	47.0	47.0
**Asia**			
Indonesia	77.0	78.0	78.0
Philippines	73.0	71.0	72.0
Thailand	54.0	49.0	51.0
Syria	35.0	43.0	42.0

Source: Based on data from ILO, 2002. No data for high income countries were available.

^a^ Income country groups – based on World Bank classification <http://web.worldbank.org/WBSITE/EXTERNAL/DATASTATISTICS/0,,contentMDK:20421402~pagePK:64133150~piPK:64133175~theSitePK:239419,00.html

^b^ Informal employment: estimated by the difference between the total non-agricultural workforce and the number of formal employees, which are those who work in corporations, quasi corporations or legally registered firms. Military and civil servants are not included.

A few studies addressed potential exposures to carcinogens among informal workers and **workplaces from informal firms.** In a study carried out in Zimbabwe using self-reported data and records from inspections, chemical substances were found in approximately 40% of firms, either formal or informal. The most common substances were solvents, cutting fluids, and dyes in urban firms, and pesticides/fertilizers in rural areas. Approximately 78.8% of workplaces were outdoors, and exposure to sunlight was common, particularly among workers from rural informal home-based firms (94%) and other rural workplaces (72%) [10]. In Dar es Salaam, Tanzania, Rongo et al. [11] analyzed workers from informal firms and observed that all welders and wood workers reported direct sunlight exposure, and over 90% perceived fumes and dusts in their workplaces. There are also reports of inappropriate management of waste or toxic substances that can affect the environment and nearby residents [12,13]. Informal firms frequently use second-hand or old-fashioned equipments that are more likely to be unsafe [10]. Personal protective equipments are rarely provided (5%) and are poorly maintained [10] or used [11].

It is important to highlight the existence of carcinogens in the workplaces where individuals share spaces and stay for long hours. In informal firms, the workplace is usually the worker’s own home [14]**,** and children or pregnant women may be at increased risk of exposure to hazardous substances. Control of exposures in the workplaces can be part of collective hazard prevention or health promotion strategies integrated into family health programs, or primary health care. Apparently this is an alternative and feasible way, given the lack of occupational health and safety services for informal workers. A recent review of literature shows that inhalation exposures in workplaces are consistently declining in time, with few exceptions [15]. However the studies under analysis were **limited to formal workers in developed** countries. Interestingly, among the reasons alleged to explain this decreasing trend, most authors mentioned regulatory actions [15,16] which unfortunately cannot be fully applied to informal economy settings.

We were unable to find studies on occupational cancers focusing on the type of employment, in order to identify precarious employment conditions or informal jobs. Based on a national representative sample survey carried out annually in Brazil, cancer was more likely to be reported by informal part-time workers (OR=3.4; 95% CI: 1.49 -7.93), but not by informal full-time workers (OR=1.2; 95% CI: 0.51 -2.82), as compared to full-time **formal** workers, in 2003. Nevertheless, no statistically significant associations were found in a previous study [17]. The identification of work-relatedness, a task commonly delegated to occupational physicians, is required when compensation differs for occupational or non-occupational diseases and injuries in the context of social security claims. In addition, in most countries only insured workers, i.e., workers having formal jobs, are eligible to obtain such compensation. Even when population-based cancer registries are available, no relevant information on occupation is usually registered, so it is not possible to relate occupation to cancer.

Estimates of the cancer burden related to occupational carcinogen exposure are mostly needed and the effort of researchers to develop global, regional and country estimates based on weak and incomplete data is notable [18].

## Competing interests

The authors declare that they have no competing financial or non-financial interests.
